# Supplementary material for: Causal Relationship between Aging and Anorexia Nervosa: A White-Matter-Microstructure-Mediated Mendelian Randomization Analysis
Source: Biomedicines. 2024 Aug 16;12(8):1874. doi: 10.3390/biomedicines12081874 (PMC11351342; doi:10.3390/biomedicines12081874)
Supplement: Supplementary file 1 [file biomedicines-12-01874-s001.zip › Supplementary Figure/Figure S9/Leaveoneout plot of MR analysis for ubm-b-1470 to AN.pdf]

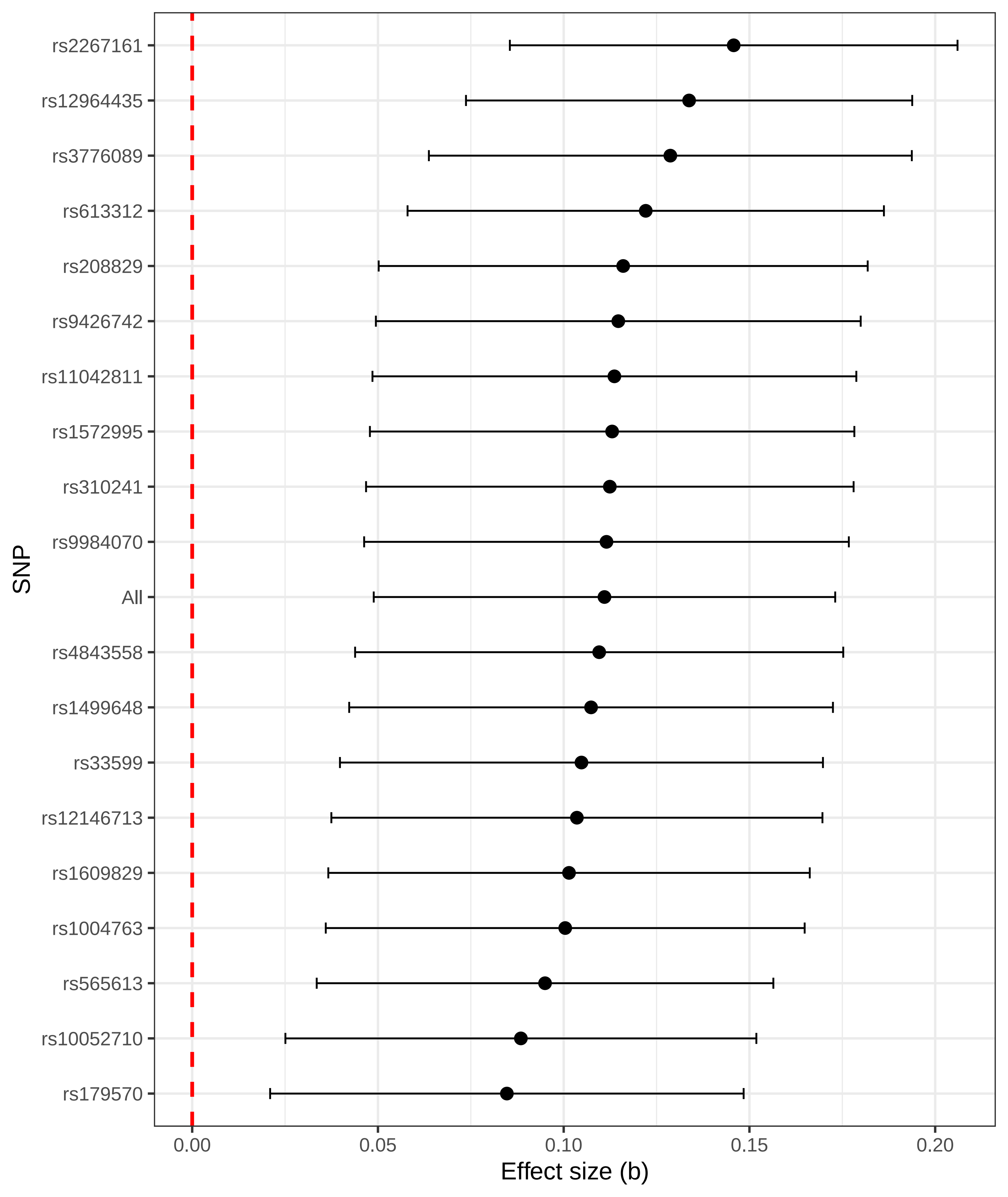

rs2267161
rs12964435
rs3776089
rs613312
rs208829
rs9426742
rs11042811
rs1572995
rs310241
rs9984070
All
rs4843558
rs1499648
rs33599
rs12146713
rs1609829
rs1004763
rs565613
rs10052710
rs179570
SNP
0.00
0.05
0.10
0.15
0.20
Effect size (b)
